# Efficacy and Tolerability Outcomes of a Phase II, Randomized, Open-Label, Multicenter Study of a New Water-Dispersible Pediatric Formulation of Dihydroartemisinin-Piperaquine for the Treatment of Uncomplicated Plasmodium falciparum Malaria in African Infants

**DOI:** 10.1128/AAC.00596-17

**Published:** 2017-12-21

**Authors:** Nicola Gargano, Lola Madrid, Giovanni Valentini, Umberto D'Alessandro, Tinto Halidou, Sodiomon Sirima, Antoinette Tshefu, Ali Mtoro, Samwel Gesase, The Eurartesim Dispersible Study Group, Quique Bassat

**Affiliations:** a Sigma-Tau Industrie Farmaceutiche Riunite S.p.A., Rome, Italy; b Centro de Investigação em Saúde de Manhiça, Maputo, Mozambique; c ISGlobal, Barcelona Centre for International Health Research (CRESIB), Hospital Clinic of Barcelona, Universitat de Barcelona, Barcelona, Spain; d Medical Research Council Unit, The Gambia, The Gambia; e Centre Muraz Bobo-Dioulasso, Nanoro, Burkina Faso; f Centre National de Recherche et de Formation en Paludisme, Ouagadougou, Burkina Faso; g Kinshasa School of Public Health, University of Kinshasa, Kinshasa, Democratic Republic of the Congo; h Ifakara Health Institute, Bagamoyo, Tanzania; i National Institute for Medical Research, Korogwe, Tanzania; j ICREA, Catalan Institution for Research and Advanced Studies, Barcelona, Spain; k Pediatric Infectious Diseases Unit, Pediatrics Department, Hospital Sant Joan de Déu (University of Barcelona), Barcelona, Spain; l Universidad Europea de Madrid, Madrid, Spain

**Keywords:** Africa, antimalarial agents, dihydroartemisinin-piperaquine, infants, malaria

## Abstract

Artemisinin combination therapies are considered the mainstay of malaria treatment, but pediatric-friendly formulations for the treatment of infants are scarce. We sought to evaluate the efficacy and safety of a new dispersible-tablet formulation of dihydroartemisinin/piperaquine phosphate (DHA/PQP) in comparison to the marketed tablet (Eurartesim) in the treatment of infants with uncomplicated Plasmodium falciparum malaria. Reported here are the results of a large phase II, randomized, open-label, multicenter trial conducted in African infants (6 to 12 months of age) from Mozambique, Burkina Faso, The Gambia, the Democratic Republic of the Congo, and Tanzania. Primary efficacy endpoint was the PCR-corrected adequate clinical and parasitological response (ACPR) at day 28. Analysis was performed for the intention-to-treat (ITT) and per-protocol (PP) populations. A total of 201 patients received the dispersible-tablet formulation, and 99 received the conventional one administered as crushed tablets. At day 28, the PCR-corrected ACPRs were 86.9% (ITT) and 98.3% (PP) in the dispersible-tablet group and 84.9% (ITT) and 100% (PP) in the crushed-tablet group. At day 42, these values were 85.9% (ITT) and 96.5% (PP) in the dispersible-tablet group and 82.8% (ITT) and 96.4% (PP) in the crushed-tablet group. The comparison between survival curves for time to new infections showed no statistically significant differences (*P* = 0.409). The safety and tolerability profile for the two groups was similar in terms of type and frequency of adverse events and was consistent with that expected in African infants with malaria. A standard 3-day treatment with the new dispersible DHA/PQP formulation is as efficacious as the currently used tablet in African infants and has a comparable safety profile. (This trial was registered at ClinicalTrials.gov under registration no. NCT01992900.)

## INTRODUCTION

The last 15 years have witnessed impressive advances in the global fight against malaria, including in sub-Saharan Africa, where the highest burden of morbidity and mortality is still concentrated (1, 2). However, an estimated 429,000 malaria-attributable deaths still occurred in 2016, the vast majority of which were among African children, making it clear that major efforts are still required in the fight against this disease (3). One of the major threats in the global strategy against malaria is posed by the emergence and potential spread of drug-resistant parasites. In response to this, the World Health Organization (WHO) recommended in 2006 the use of artemisinin-based combination therapies (ACTs) for the treatment of uncomplicated malaria (4). The artemisinin derivatives are currently the most rapidly acting and potent antimalarials (5). The pharmacodynamic effects of ACTs are due to the rapid absorption of artemisinins and their strong activity against several stages of the malaria life cycle from young asexual forms (rings) to early sexual forms (gametocytes) (6).

Eurartesim is a fixed-dose combination composed of dihydroartemisinin (DHA) and piperaquine phosphate (PQP) (7). This partner compound has a different mechanism of action from that of artemisinins and a much longer half-life (several weeks), facilitating the initial clearing of parasites, and a long posttreatment prophylactic effect (8, 9).

The efficacy and safety of Eurartesim for the treatment of uncomplicated Plasmodium falciparum malaria has been demonstrated in two phase III trials, respectively conducted in 1,553 African children and 1150 Asian adults and children (10, 11). Both studies demonstrated an equivalent efficacy of DHA/PQP versus the comparative ACT. In addition, they evidenced a superiority of DHA/PQP versus the comparative drugs in a longer posttreatment prophylaxis period that suppress new infections from emerging during follow-up.

In 2011, Eurartesim obtained a marketing approval by the European Medicine Agency for all countries of the European Union, manufactured as film-coated tablets (12). The ACT was marketed for its use in children and adults and has been adopted by several countries where malaria is endemic as the first-line treatment of uncomplicated malaria (3). Finally, Eurartesim obtained by the WHO the prequalification status on 9 October 2015 (13).

One of the greatest challenges of malaria treatment is ensuring adequate intake and compliance among young children, the population group most at risk of developing life-threatening episodes (14). As of today, very few antimalarials are available in good laboratory practice (GLP)-produced pediatric friendly formulations (15). Since infants and very young children are generally unable to swallow oral tablets, Eurartesim was administered in previous trials as an oral suspension of crushed tablets mixed with water. However, the bitter taste of the crushed tablet could easily compromise tolerability in this age group, and crushing tablets is a suboptimal preparation procedure, which could result in loss of drug and a reduced dose ingested. To overcome these problems, a new water-dispersible tablet formulation of DHA/PQP was developed for oral administration in infants and young children.

We present here the results of a multicenter trial in sub-Saharan Africa aiming to assess the efficacy, tolerability and safety of the new dispersible-tablet formulation of DHA/PQP with respect to the marketed formulation, administered as a crushed tablet, to infant patients (from 6 to 12 months of age) with uncomplicated P. falciparum malaria. These results are part of a large phase II trial, which was designed to also collect pharmacokinetic data for dispersible and crushed formulations of DHA/PQP in African infants with malaria.

## RESULTS

**FIG 1:**
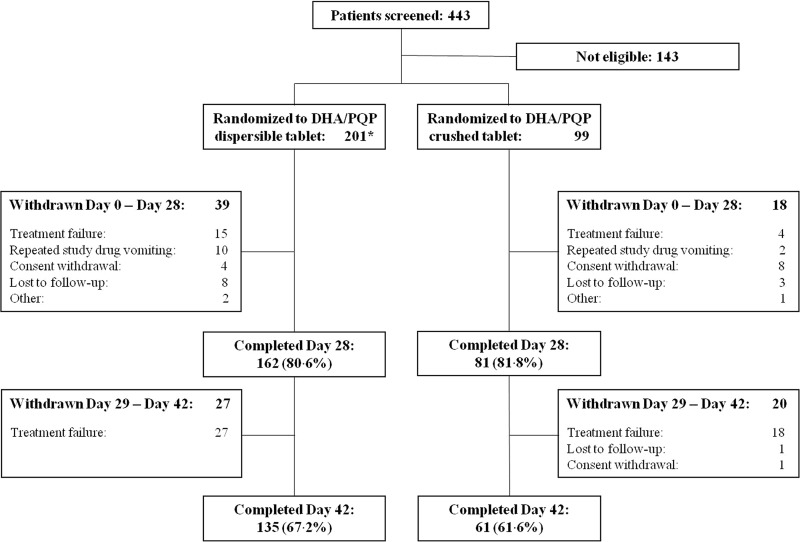
Flow chart of study participants with uncomplicated P. falciparum malaria treated with a 3-day course of either dispersible or crushed tablets of dihydroartemisinin-piperaquine (DHA/PQP). *, Two patients randomized in the dispersible treatment group were excluded from all analysis due to unconfirmed malaria diagnosis at day 0 (classified as “Other”) and informed consent withdrawal before first study drug administration.

Patient disposition as presented in the case report forms is summarized in Fig. 1. Overall, 443 patients were screened, and 300 were randomized: 201 to receive the dispersible-tablet formulation and 99 to receive the crushed-tablet formulation. However, two patients randomized in the dispersible-tablet group were excluded by all analysis because one of them did not have parasites at day 0 (as determined by a confirmatory blood smear reading) and the other withdrew informed consent before the first drug administration (these patients, respectively, are reported as “other” and “consent withdrawal” by day 28 in Fig. 1).

The most common reasons for study withdrawal were malaria recurrence (classified as treatment failure) and informed consent withdrawal, occurring with a similar frequency in both treatment groups. Repeated vomiting after drug administration was more frequent in the dispersible arm compared to the crushed arm (5% versus 2%), although such a difference was not statistically significant (*P* = 0.349 by the Fisher exact test). Among patients reported as “other” in Fig. 1, one more patient in the dispersible-tablet group dropped out due to family movement to a another area, while one patient in the crushed-tablet group left the hospital after the first study drug intake.

The number of patients enrolled in each country and their attribution to ITT and PP populations are shown in Table 1. The patients excluded from the PP population due to major protocol violations were 28 in the dispersible-tablet group (including the two patients above described, who were also excluded from the ITT population) and 15 in the crushed-tablet group (Table 2). All of them fulfilled the *a priori* declared major violations or did not complete the study due to a reason different from malaria recurrence or any other AE causing withdrawal from the study (i.e., consent withdrawal or lost to follow-up). Two more patients were excluded from the PP population (one per each treatment group) since it was not possible to assess their outcome on day 28 not having the patients attended this visit and presenting malaria recurrence at day 42. There were also seven randomized patients presenting baseline parasitemia outside the range indicated in inclusion criteria, but all of these violations were judged as “minor” by the Data Safety Monitoring Board (DSMB), and according to the study protocol these patients were included in the PP population. Overall, there was a good balance for demographic and baseline characteristics between treatment arms (Table 3).

**TABLE 1:** Patient accountability by country in ITT and PP populations^*a*^

Study population	DHA/PQP dispersible-tablet group				DHA/PQP crushed-tablet group			
	ITT		PP		ITT		PP	
	*n*	%	*n*	%	*n*	%	*n*	%
Total	199	100	173	100	99	100	84	100
Mozambique	90	45.2	83	48.0	51	51.5	40	47.6
Gambia	11	5.5	6	3.5	3	3.0	3	3.6
DR-Congo	26	13.1	25	14.5	7	7.1	7	8.3
Burkina Faso	67	33.7	57	33.0	37	37.4	33	39.3
Tanzania	5	2.51	2	1.2	1	1.0	1	1.2

a *n*, number of patients. DR-Congo, Democratic Republic of the Congo.

**TABLE 2:** Summary of major protocol violations

Category	DHA/PQP treatment group	
	Dispersible tablet (*n* = 199)	Crushed tablet (*n* = 99)
Total no. of patients with major violations	26	15
% of patients with major violations	13.1	15.2
Protocol violation, no. (%) of patients		
Repeated study drug vomiting	10 (5.0)	2 (2.0)
Study drug noncompliance	2 (1.0)	0
Presence of jaundice at screening	1 (0.5)	0
Consent withdrawal	3 (1.5)	8 (8.0)
Visit at day 28 not performed and malaria recurrence at day 42	1 (0.5)	1 (1.0)
Lost to follow-up at or before day 42	8 (4.0)	3 (3.0)
Moved away from the study site	1 (0.5)	0
Left the hospital after first drug administration	0	1 (1.0)

**TABLE 3:** Demographic and baseline characteristics (ITT population)

Characteristic	DHA/PQP treatment group	
	Dispersible tablet (*n* = 199)	Crushed tablet (*n* = 99)
No. (%) of patients		
Male	89 (44.7)	47 (47.5)
Female	110 (55.3)	52 (52.5)
Mean age and wt ± SD		
Age (mo)	9.1 ± 1.8	9.2 ± 2.0
Wt (kg)	7.9 ± 1.2	8.0 ± 1.1
Clinical data		
Fever, no. (%) of patients	119 (59.8)	61 (61.6)
Mean temp in °C (range)	37.8 (35.8–40.3)	37.8 (35.8–40.2)
Mean parasite density (range)	53,282 (771–226,707)	56,480 (108–322,194)
Presence of gametocytes, no. (%) of patients	10 (5.0)	5 (5.1)
Mean hemoglobin in g/dl (range)	8.96 (5.40–12.00)	8.98 (6.90–12.40)

### Efficacy outcomes.

**FIG 2:**
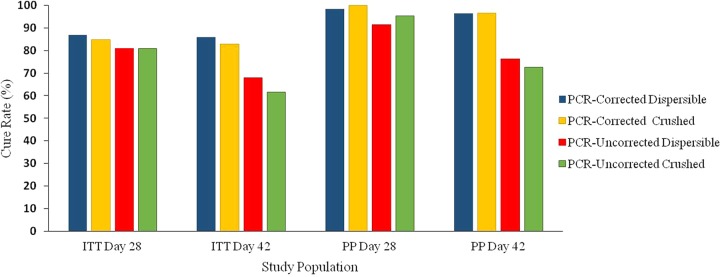
ACPR at days 28 and 42 of treatment with dispersible and crushed DHA/PQP formulations administered to infant patients with uncomplicated P. falciparum malaria.

Both dispersible and crushed formulations were efficacious. Descriptive and inferential statistics for the cure rates are shown in Table 4 and efficacy results are summarized in Fig. 2. In the PP population, the PCR-corrected adequate clinical parasitological responses (ACPRs) at day 28 were very high for both treatment arms (98.3 and 100% for the dispersible and crushed groups, respectively), showing homogeneity among cure rates with overlapping two-sided asymptotic confidence intervals (CI). Similarly, the PCR-uncorrected ACPR was comparable between treatment arms and still above 90% for both groups. At day 42, the PCR-corrected ACPRs were slightly lower than that observed at day 28 but remained very similar among treatment arms (96.5 and 96.4% for the dispersible and crushed groups, respectively), whereas the uncorrected ACPRs for the dispersible arm were to some extent higher with respect to the crushed arm (76.3% versus 72.6%).

**TABLE 4:** PCR-corrected and uncorrected adequate clinical and parasitological response by time points in ITT and PP populations

Study population^*b*^	Cure rate (ACPR)	No. (%) of patients in DHA/PQP treatment group		Treatment difference (DHA/PQP dispersible–DHA/PQP crushed)		
		Dispersible table	Crushed tablet	Δ (%)	Two-sided 95% CI^*a*^	
					Without CC	With CC
Day 28						
ITT (*n* = 199, 99)	PCR corrected	173 (86.9)	84 (84.9)	2.08	−0.064–0.106	−0.071–0.113
	Uncorrected	161 (80.9)	80 (80.8)	0.09	−0.094–0.096	−0.101–0.103
PP (*n* = 173, 84)	PCR corrected	170 (98.3)	84 (100)	−1.73	−0.037–0.002	−0.046–0.011
	Uncorrected	158 (91.3)	80 (95.2)	−3.91	−0.101–0.023	−0.110–0.032
Day 42						
ITT (*n* = 199, 99)	PCR corrected	171 (85.9)	82 (82.8)	3.10	−0.058–0.120	−0.065–0.127
	Uncorrected	135 (67.8)	61 (61.6)	6.22	−0.053–0.178	−0.061–0.186
PP (*n* = 173, 84)	PCR corrected	167 (96.5)	81 (96.4)	0.10	−0.047–0.049	−0.056–0.058
	Uncorrected	132 (76.3)	61 (72.6)	3.68	−0.078–0.151	−0.087–0.160

a CI, confidence interval; CC, continuity correction.

b Numbers in parentheses correspond to the numbers of individuals treated with the dispersible tablet and the crushed tablet, respectively.

In the ITT population, the PCR-corrected ACPRs at day 28 were indeed similar between treatment arms (86.9 and 84.9% for the dispersible and crushed groups, respectively), while the uncorrected ACPRs were nearly identical (80.9 and 80.8%). At day 42, the PCR-corrected ACPRs were to some extent lower than those observed at day 28 for both arms (Fig. 2). Similar to the findings observed in the PP population, the uncorrected ACPRs in the ITT population for the dispersible-tablet group were slightly higher with respect to the cure rate for the crushed-tablet group. Overall, for both PCR-corrected and uncorrected ACPRs, the asymptotic 95% CI values showed a good homogeneity with overlapping values and including the zero value for the computed differences (Table 4). Considering both ITT and PP populations overall, the comparison between the dispersible and crushed groups for the corrected and uncorrected ACPRs, assessed at day 28 or 42, showed no statistically significant differences.

**FIG 3:**
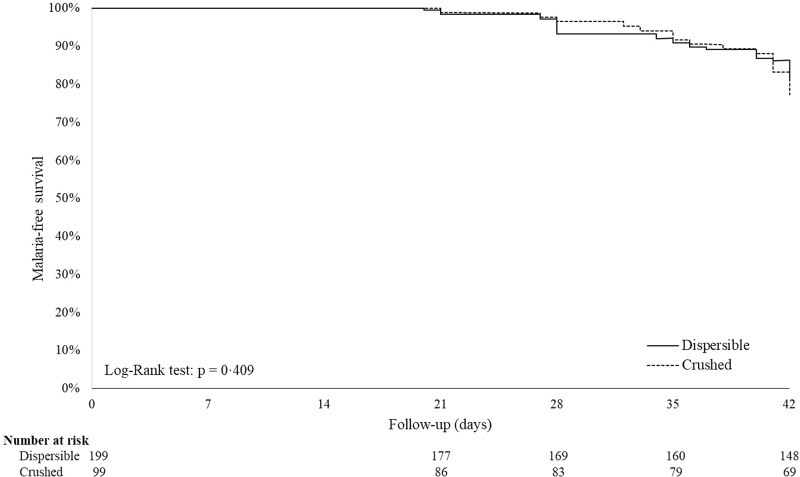
Time to new infections: survival analysis. A Kaplan-Meyer curve shows the cumulative risk of developing new infections in infants (ITT population) treated with either a dispersible- or a crushed-tablet formulation during a 42-day follow-up period.

The occurrence of malaria recurrences by time points, as well as other reasons for treatment failure, are shown in Table 5. At day 28, the numbers of new infections were similar between the treatment arms (6 and 4% for the dispersible and crushed groups, respectively), whereas only two recrudescences occurred in the dispersible-tablet group. By day 42, the proportion of patients experiencing new clinical malaria episodes increased similarly in both dispersible and crushed groups (ITT population), reaching, respectively, 18.1 and 21.2% of the new infections (*P* = 0.519 [chi-square test]) and 2.5 and 1.0% of recrudescences (*P* = 0.667 [Fisher exact test]). When estimated by Kaplan-Meier survival analysis, the cumulative risks of new infection at day 42 were 19.8 and 23.6% for the dispersible and crushed groups, respectively (Fig. 3), whereas those for recrudescence were 3.1 and 1.3% (ITT population). Similar results were obtained for the PP population (data not shown). As for the cure rates, comparison by log-rank tests between treatment groups on time to new infection and time to recrudescence was not statistically significant in both ITT and PP populations (i.e., new infections, *P* = 0.409 and *P* = 0.568, respectively; recrudescences, *P* = 0.404 and *P* = 0.162, respectively).

**TABLE 5:** Treatment failure by time point (ITT population)

Reason for failure	No. and % of patients in each treatment group							
	Day 28				Day 42			
	Dispersible tablet (*n* = 199)		Crushed tablet (*n* = 99)		Dispersible tablet (*n* = 199)		Crushed tablet (*n* = 99)	
	*n*	%	*n*	%	*n*	%	*n*	%
Recrudescence^*a*^	2	1.0	0	0.0	5	2.5	1	1.0
New P. falciparum infection	12	6.0	4	4.0	36	18.1	21	21.2
Early treatment failure (ETF)	1	0.5	0	0.0	1	0.6	0	0.0
Late treatment failure (LTF)	14	7.0	4	4.0	41	20.6	22	22.2
Late clinical failure (LCF)	3	1.5	2	2.0	18	9.1	12	12.1
Late parasitological failure (LPF)	11	5.5	2	2.0	23	11.6	10	10.1
Lost to follow-up	8	4.0	3	3.0	8	0.0	4	1.0
Repeated vomiting	10	5.0	2	2.0	10	5.0	2	2.0
Withdrawal before the time of analysis (day 28): any reason except lost to follow-up	4	2.0	9	9.1	4	2.0	9	9.1
Day 28 visit missing and parasitemia at day 42	1	0.5	1	1.0	-	-	-	-
Adverse event	0	0.0	0	0.0	0	0.0	1	1.0
Total no. of failures	38	19.0	19	19.2	64	32.2	38	38.4

a For two patients (one for each group) with malaria recurrence, PCR analysis was not performed. Therefore, DSMB categorized these patients as treatment failures in the ITT population and missing in the PP population.

One ETF occurred during the study due to the evolution of uncomplicated into severe malaria in a patient enrolled 2 days after initial malaria symptoms. The complication occurred 20 h after the first study drug intake and was probably due to the late starting of antimalarial treatment. At day 42, the overall proportions of treatment failures (ETFs plus LTFs) were similar between the treatment arms, corresponding to 42 cases (21.1%) in the dispersible-tablet group and 22 cases (22.2%) in the crushed-tablet group (*P* = 0.825 [chi-square test]; ITT population).

Parasite clearance was rapid in both treatment arms. In the ITT population, fewer than half of the patients were parasitemic at day 1 (46.2 and 48.5% in the dispersible and crushed groups, respectively), with only few of them remaining parasitemic at day 2 (3.0% versus 2.0%). With the exception of one patient who was still parasitemic up to day 7 (dispersible-tablet group), all other patients were aparasitemic by day 3. No statistically significant differences in parasitemia clearance between treatment groups were found using the log-rank test (*P* = 0.653).

At baseline, about 60% of the patients had fever. At day 2, more than 96% of the patients were afebrile in both groups. Upon enrollment, only 5% of the patients in both groups presented gametocytes, which quickly disappeared in the first few days. Gametocytes did not develop during the course of the study. Due to the quick disappearance of fever and the gametocytes, the corresponding survival analyses were not performed.

### Safety results.

Both DHA/PQP formulations were well tolerated, with most AEs of mild or moderate severity. The AE profiles for both treatment groups were similar in terms of the type and frequency of events. Totals of 160 (80.4%) and 84 (84.9%) patients, respectively, treated with the dispersible or crushed formulations, had at least one AE during the study. Anemia, vomiting, diarrhea, rhinitis, and cough were the most frequently reported AEs and were often associated with malaria, influenza, and respiratory tract infections. The occurrence of laboratory AEs, e.g., altered liver enzymes (AST), was very rare and was similar between the treatment groups.

The proportion of patients experiencing at least one treatment-emergent adverse event (TEAE), for which the relationship with the study drug was suspected, was slightly more prevalent in patients treated with the crushed-tablet formulation than in patients treated with the dispersible-tablet formulation (42.4% versus 33.7%, *P* = 0.139 [chi-square test]) (Table 6). In particular, gastrointestinal tolerability was better for patients treated with the dispersible-tablet formulation since vomiting occurred less frequently in this group compared to the crushed-tablet group (22.6% versus 31.3%, *P* = 0.105 [chi-square test]). Cutaneous reactions were infrequent, with only one case of rash (crushed group). One patient in the dispersible-tablet group developed moderate anemia, which occurred 1 week after treatment and resolved 3 weeks later. Overall, none of the TEAEs required hospitalization.

**TABLE 6:** Treatment-emergent adverse events suspected to be related to the study treatment administration (ITT population)

System organ class and preferred term	No. and % of patients in each DHA/PQP treatment group			
	Dispersible tablet (*n* = 199)		Crushed tablet (*n* = 99)	
	*n*	%	*n*	%
Patient with at least one TEAE^*a*^	67	33.7	42	42.4
Blood and lymphatic system disorders				
Anemia	1	0.5	0	0.0
Thrombocytosis	1	0.5	0	0.0
Gastrointestinal disorders				
Diarrhea	0	0.0	2	2.0
Vomiting	45	22.6	31	31.3
Nausea	1	0.5	0	0.0
Salivary hypersecretion	3	1.5	1	1.0
General disorders and administration site conditions				
Pyrexia	0	0.0	1	1.0
Infections and infestations				
Gastroenteritis	1	0.5	2	2.0
Malaria	1	0.5	0	0.0
Investigations				
Electrocardiogram QTcF prolonged (>60 ms)	23	11.6	15	15.2
Metabolism and nutrition disorders				
Decreased appetite	1	0.5	1	1.0
Respiratory, thoracic, and mediastinal disorders				
Cough	1	0.5	0	0.0
Skin and subcutaneous tissue disorders				
Papulosquamous rash	0	0.0	1	1.0

a TEAE, treatment-emergent adverse event.

There were no significant differences between treatment groups in ECG parameters at baseline and during follow-up. A relevant and comparable reduction of HR (heart rate) in both treatment groups was observed at day 2 and at day 7. Related with this reduction, an increase in PR, QT, and QTcF intervals was observed in both groups with a comparable extension. A change in QTcF interval from baseline was detectable at day 2 predose and increased after drug administration, with 22 patients in the dispersible-tablet group (11.1%) and 14 patients in the crushed-tablet group (14.1%) manifesting a QTcF prolongation over 60 ms, none of which included clinical translation. No cases of QTcF prolongation higher than 500 ms were reported. At day 7, the QTcF prolongation tended to normalization, with only one patient in the dispersible-tablet group and two patients in the crushed-tablet group still maintaining QTcF prolongation. Overall, no arrhythmias or any other cardiovascular AEs were reported.

Two severe adverse events (SAEs) occurred during the study, both judged unrelated to the study treatment. The first SAE involved the death of a 6-month-old male patient enrolled in the crushed-tablet group. He received the full treatment course and was discharged from the hospital after malaria resolution (day 3). At day 27, the child was seen as an outpatient in a peripheral health post, with a history of fever, cough, and diarrhea. After a positive rapid diagnostic test for malaria, he was treated with artemether/lumefantrine. However, a malaria diagnosis was not confirmed by microscopy, and 2 days later the mother reported the child's death. The investigator considered sepsis the most likely cause of death (a diagnosis supported by a verbal autopsy). The second SAE occurred in a male of 11 months enrolled in the dispersible-tablet group. He received the first dose of the study drug, and 20 h later the patient condition deteriorated, with evidence of severe anemia (hemoglobin, 4.9 g/dl) and polypnea. The study treatment was interrupted, and the patient received parenteral quinine for malaria and blood transfusion for anemia. After 4 days of hospitalization, followed by oral treatment with quinine for a further 5 days, the event was resolved.

## DISCUSSION

According to guidelines on the clinical investigation of medicinal products in a pediatric population, there is a need to develop new formulations that allow accurate dosing and enhance patient compliance in infants and young children (16). This recommendation becomes even more necessary for the treatment of malaria, as young children and infants are those most affected by the disease, and very few GMP-produced pediatric-friendly formulations are available (15). Artemisinin-based combination therapy (ACT) is the current standard of care for patients with uncomplicated malaria in Africa (4). ACTs are typically provided in tablet form, which can be challenging to administer to young children who are typically unable to swallow whole pills. To facilitate the administration of ACTs to these individuals, tablets can be crushed and mixed with water. However, this process can result in the loss of active ingredients and lead to underdosing. In addition, crushed antimalarial tablets can be unpalatable since they have a bitter taste causing children to spit them out, thus leading to uncertain and/or subtherapeutic dosing (17).

Presently, there is only one ACT reported in the last updated version of the WHO Model List of Essential Medicines for Children (last amended on August 2015), containing arthemether-lumefantrine, which was specifically developed as a dispersible formulation to improve the effectiveness and accuracy of ACT dosing in infants and young children (15). Thus, there is an urgent need to make available on the market other pediatric formulations of different ACTs and, in this context, a new dispersible formulation of the DHA/PQP fixed-dose combination was developed for oral administration.

The dispersible-tablet formulation was similar in tolerability, efficacy, and safety to the standard formulation administered as crushed tablet to infants with uncomplicated P. falciparum malaria. In particular, PCR-corrected cure rates at day 28 of follow-up were high for both formulations, and no differences were observed in the response to treatment in terms of clearance of asexual parasites and fever, which were rapid in both treatments groups. Follow-up periods longer than 28 days are currently recommended by the WHO for antimalarial drugs with a long half-life (e.g., piperaquine or mefloquine) to allow drug concentrations in the blood to fall below the minimum therapeutic threshold (25). In fact, short observation periods can yield an underestimation of recrudescence rates. Hence, analysis of cure rates on day 42 showed that they were still high, suggesting a sustained efficacy for both formulations. Furthermore, substantial differences between the efficacies of the two formulations were not observed for the uncorrected ACPRs at day 42. Because the uncorrected cure rate is mainly affected by new infections in high-transmission areas, this finding indicates a similar prophylactic effect exerted by both formulations.

The present study confirmed the satisfactory efficacy of Eurartesim previously obtained in phase III trials performed in Africa and Asia (7). In particular, the PCR-corrected ACPR at day 28 or the incidence of recurrences were similar to that obtained in African children (10).

The dispersible formulation was well tolerated, and its safety profile was comparable to the crushed tablet. No new safety issues arose from the present study, and all findings were in line with the former ones. Most of the commonly reported AEs were symptoms of malaria. Similarly, the pattern of changes in laboratory variables was consistent with acute malaria and its resolution, with no differences between treatment groups. The most common drug-related AE was vomiting, and the corresponding frequency was similar to that previously reported in African children treated with crushed or uncrushed tablets (10). Importantly, no arrhythmias or other cardiovascular events were reported during the study. QTcF interval prolongations (difference from baseline higher than 60 ms) were observed in 11.6 and 15.2% of patients in the dispersible and crushed groups, respectively (*P* = 0.381 [chi-square test]). Prolongation of the QT interval was assessed using both Fridericia's and Bazett's correction methods but, notably, both formulas could be biased by the physiological high HR present in infants and exacerbated by the ill status and fever. In fact, baseline evaluations differed drastically (by ∼60 ms) when the different correction methods were applied. The reduction in HR observed after drug administration could also induce a bias in the estimation of QTc. Accordingly, QTcF recorded at the screening visit was lower than that recorded at day 7 (335 ± 16 ms versus 410 ± 22 ms), when malaria was resolved and the blood level of piperaquine was below the concentration considered relevant for sustaining QT interval prolongation. These considerations suggest that the QTcF assessment in infants by DHA/PQP could be overestimated. Nonetheless, QTcF never exceeded the normal limits (450 to 470 ms), no cases evolved in rhythm disturbance, and all of the changes observed during therapy were never associated with clinical signs of cardiotoxicity.

Dispersible tablets are expected to contribute to ease of drug administration in subjects having difficulties in swallowing whole tablets, i.e., infants and young children. Since the dispersible formulations were similar in efficacy and safety to the conventional formulation in the infant population, we believe that this highly efficacious formulation should be made more readily available to all children below 5 years of age. Cost savings are also likely with its use, with the potential benefit of improving the acceptability of the combination once available on the market. It is noteworthy that a recent cost-effectiveness analysis of DHA/PQP for first-line treatment of uncomplicated malaria in African children showed that the use of this ACT, particularly in areas with moderate to high malaria transmission, is clearly justifiable from both clinical and economic perspectives (18, 19).

The results presented here are part of a large study that was designed to evaluate also the population pharmacokinetics of piperaquine and dihydroartemisinin in African infants, since it appears important to demonstrate bioequivalence of the new formulation on PK grounds once similar efficacy and safety have been confirmed. The new dispersible formulation of Eurartesim is easy and safe to administer and provides better grounds for an enhanced compliance and effective treatment; hence, this should facilitate drug registration by Regulatory Authorities, prequalification by the WHO and, finally, full adoption in malaria control programs. Further studies are needed to clearly establish the improvement of the new formulation on compliance, hoping that the simplicity of its administration will improve substantially the adherence to the full 3-day course of treatment, therefore impacting the reduction of malaria morbidity and mortality in infants and young children and minimizing parasite resistance to ACTs.

## MATERIALS AND METHODS

### Study design.

Between November 2013 and June 2015, a phase II, randomized, open-label, multicenter trial was performed in seven sites of five African countries: Centro de Investigação em Saude da Manhiça, Maputo, Mozambique; Kinshasa School of Public Health, University of Kinshasa, Democratic Republic of the Congo; Centre Muraz Bobo-Dioulasso, Nanoro, Burkina Faso; Centre National de Recherche et de Formation en Paludisme, Ouagadougou, Burkina Faso; Ifakara Health Institute, Bagamoyo, Tanzania; National Institute for Medical Research, Korogwe, Tanzania; and Medical Research Council Unit, The Gambia.

The study protocol was approved by the Institutional Review Board of the Hospital Clínic of Barcelona (Spain) and by the National Ethics Review Committee and/or Institutional Review Board at each trial site. The trial was conducted under the provisions of the Declaration of Helsinki and in accordance with GCP guidelines (20, 21). A data safety monitoring board (DSMB) was established, working independently, to harmonize and monitor patients' safety. The trial was registered in the ClinicalTrial.gov registry under registration no. NCT01992900 on 4 September 2014.

### Patients.

A targeted number of 300 infants (male and female) with uncomplicated malaria were enrolled in the study. Since the study was designed to evaluate also the population pharmacokinetics of piperaquine and dihydroartemisinin in African infants, the sample size has been estimated on the basis of previous PK studies (22, 23), and all efficacy and safety analyses are descriptive, including the *P* values and confidence intervals. The inclusion criteria were age 6 to 12 months, a body weight of ≥5 kg, a P. falciparum monoinfection with asexual parasite densities between 1,000 and 200,000 parasites/μl of blood, fever (axillary temperature of ≥37.5°C), or a history of fever in the preceding 48 h. Exclusion criteria were previous treatment with antimalarials, acute malnutrition, severe malaria, danger signs, moderate/severe anemia (Hb < 7 g/dl), a family history of sudden death or known congenital prolongation of the QT interval, or treatment with QT prolongation inducers or strong cytochrome-P450 inhibitors/inducers or antiretroviral drugs (or lactated by HIV-positive women under antiretroviral therapy). Patients satisfying the inclusion and exclusion requirements were enrolled if the parent or guardian signed a written informed consent.

### Randomization and masking.

Randomization was centralized using an interactive web-based response system. A randomization list was generated by an independent contract research organization (CROS NT, Italy), with each treatment allocation concealed in sealed opaque envelopes that were opened by the investigators only after patient randomization. An allocation ratio of 2:1 (dispersible versus crushed formulation) was applied, balancing patients for sex, to recruit 200 patients to receive the dispersible DHA/PQP formulation and 100 patients to receive the marketed tablet. No stratification for countries or sites was applied.

### Procedures.

The DHA/PQP dispersible formulation was a coformulated, water-dispersible flat tablet, provided in two different strength dosages: 10/80 mg and 20/160 mg of dihydroartemisinin/piperaquine tetraphosphate (as cellulose-microencapsulated piperaquine tetraphosphate) and other components (cellulose, starch, croscarmellose, black cherry flavor, saccharine, sucrose, and magnesium stearate). The marketed Eurartesim formulation was a coformulated, film-coated tablet, provided in one strength of 20/160 mg of DHA/PQP (Sigma-Tau, Italy) (12). Both formulations were administered once a day for three consecutive days, according to body weight. Patients weighing 5 to 7 kg received a daily dose of 10/80 mg of DHA/PQP, while patients weighing >7 kg to <13 kg received a daily dose of 20/160 mg of DHA/PQP.

DHA/PQP administration has a food interaction effect resulting in an increase of piperaquine absorption when concomitantly administered with high-fat food (23). To reduce this effect, the first dose was administered as soon as randomization was done, and deliberate efforts were made to ensure that no food was administered in the following 3 h. For the other doses, patients should not have been fed in the 3 h before drug intake and for the following 3 h. However, for infants needing food during the restricted periods, this was limited to breast milk or a low-fat maize porridge.

Patients were kept at the health facility for the 3-day dosing period. Scheduled visits during follow-up were at days 7, 14, 21, 28, and 42 posttreatment. In case of recurrent parasitemia, rescue treatment was performed according to local guidelines.

The diagnosis of P. falciparum infection was made at each site by microscopy using standard methods (24). Blood slides collected from screening until parasitemia clearance were also read by a centralized laboratory (Centro de Investigaçao em Saúde da Manhiça, Maputo, Mozambique). If parasitemia recurred during the study, the distinction between recrudescence and new infection was made by PCR genotyping (25). PCR analysis was centralized and performed under blind conditions at the Institute of Tropical Medicine, Antwerp, Belgium. Three polymorphic markers (MSP1, MSP2, and GluRP) were used to distinguish recrudescence from new infections. Recrudescence was defined when at least one identical allele for each marker was detected in the pre- and posttreatment samples. New infections were defined when all alleles for at least one marker differed between the two samples.

Blood samples for hematology and biochemistry were taken at enrollment and at day 7 and then repeated at day 28 if clinically significant abnormalities were detected at day 7 (analysis of main hematological and biochemical parameters, assessed at days 0, 7, and 28 are summarized in Table S1 in the supplemental material).

A 12-lead electrocardiogram (ECG) was recorded for each patient at enrollment (baseline) and then repeated at day 2 before the last drug administration, as well as after 4 to 6 h of drug intake. An ECG was also recorded at day 7 and repeated at day 28 if clinically relevant abnormalities were detected at day 7. All ECGs were digitalized using an ELI-150 cardiograph (Mortara Instruments Europe Srl, Italy), and ECG reading and analysis were centralized by a cardiac laboratory (Cardiabase, Nancy, France).

### Outcomes.

The main efficacy endpoint was the PCR-corrected adequate clinical parasitological response (ACPR) at day 28. Other efficacy and safety endpoints included the following: (i) day 28, PCR-uncorrected ACPR; (ii) day 42, PCR-corrected and -uncorrected ACPR; (iii) the proportion of patients with early and late treatment failure (ETF and LTF); (iv) asexual parasite density and clearance time; (v) fever clearance time and gametocyte carriage over time; (vi) Kaplan-Maier survival analysis for new infections and recrudescences over time; (vii) AE occurrence; and (viii) changes in hematology, blood chemistry, vital signs, and ECG parameters.

Treatment outcome was assessed according to the study protocol and further revised by the DSMB to complement the WHO *in vivo* efficacy definitions (26). All cases not strictly matching these rules (e.g., patients having taken disallowed drugs or with partially missing data, such as blood parasitemia) were evaluated under blind conditions at the DSMB meetings.

### Statistical analysis.

All statistical analyses were performed using the SAS system software, version 9.2.

Analyses of efficacy were performed for both the ITT and the PP populations, whereas safety analyses were performed only for the ITT population. The ITT population included all patients taking at least one dose of the study drug. The PP population included all randomized patients who received the full treatment course, completed the day 28 assessment, had an evaluable PCR in case of recurrent parasitemia, and did not meet major protocol violations (e.g., inclusion without a microscopically confirmed diagnosis or the occurrence of severe vomiting or severe malaria). Patients for whom the PCR indicated a new infection were considered failures in the PCR-uncorrected analysis and successes in the PCR-corrected analysis, whereas patients with PCR results indicating a recrudescence were counted as failures in both analyses. Patients for whom the PCR result was not interpretable or missing or not done were imputed as failures in the ITT population and excluded in the PP population. Patients lost to follow up were excluded from the PP population (see Table S2 in the supplemental material).

The two-sided 95% confidence interval (CI) for the treatment difference in the PCR-corrected and uncorrected ACPRs at day 28 (or day 42) were generated using the normal approximation method (27). The limits of the asymptotic CIs were computed. A CI with continuity correction was also computed to verify the robustness of the conclusions. Whenever the observed counts were small (i.e., not all cells had observed counts of at least five) or there was a disagreement between the results of the two formulas (with or without continuity correction), an exact CI was computed.

The proportion of aparasitemic patients was computed for each study visit by treatment group. The parasite clearance time was defined as the time elapsed between the first study drug intake and the first of two consecutive evaluations with negative parasitemia. The proportion of afebrile patients was computed at each study visit by treatment group. Early and late treatment failures were summarized by proportion per each treatment group.

The cumulative risks of new infections and recrudescences were estimated through survival analysis (Kaplan-Meier). In survival analyses of new infections, patients classified as new infections were considered as having reached the event, whereas patients who did not reach the event (i.e., subjects withdrawing from the study or having a recrudescence or with an uninterpretable or missing PCR or classified as a success) were censored at the relevant time. In survival analyses of recrudescences, patients classified as recrudescence were considered to have reached the event, whereas patients who did not reach the event (i.e., subjects withdrawing the study or with a new infection or with an uninterpretable/missing PCR or classified as success) were censored at the relevant time.

## Supplementary Material

Supplemental material
