# Supplementary material for: Efficacy and Tolerability Outcomes of a Phase II, Randomized, Open-Label, Multicenter Study of a New Water-Dispersible Pediatric Formulation of Dihydroartemisinin-Piperaquine for the Treatment of Uncomplicated Plasmodium falciparum Malaria in African Infants
Source: Antimicrob Agents Chemother. 2017 Dec 21;62(1):e00596-17. doi: 10.1128/AAC.00596-17 (PMC5740378; doi:10.1128/AAC.00596-17)
Supplement: Supplemental material [file supp_62_1_e00596-17__index.html]

Efficacy and Tolerability Outcomes of a Phase II, Randomized, Open-Label, Multicenter Study of a New Water-Dispersible Pediatric Formulation of Dihydroartemisinin-Piperaquine for the Treatment of Uncomplicated Plasmodium falciparum Malaria in African Infants — Supplemental material 

# Efficacy and Tolerability Outcomes of a Phase II, Randomized, Open-Label, Multicenter Study of a New Water-Dispersible Pediatric Formulation of Dihydroartemisinin-Piperaquine for the Treatment of Uncomplicated Plasmodium falciparum Malaria in African Infants

## Supplemental material

- Supplemental file 1 -

  Tables S1 and S2

  PDF, 51K
