# Supplementary material for: Efficacy and Tolerability Outcomes of a Phase II, Randomized, Open-Label, Multicenter Study of a New Water-Dispersible Pediatric Formulation of Dihydroartemisinin-Piperaquine for the Treatment of Uncomplicated Plasmodium falciparum Malaria in African Infants
Source: Antimicrob Agents Chemother. 2017 Dec 21;62(1):e00596-17. doi: 10.1128/AAC.00596-17 (PMC5740378; doi:10.1128/AAC.00596-17)
Supplement: Supplemental material [file AAC.00596-17_zac001186785s1.pdf]

Supplementary Table 1. Summary of haematology and biochemistry by visit (main parameters) – ITT population

		DHA/PQP Dispersible group		DHA/PQP Crushed group	
**Parameter**	**Visit**	**N**	**median (range, min-max)**	**N**	**median (range, min-max)**
Haemoglobin (g/dL)	Day 0	199	9.0 (5.4-12.0)	99	8.8 (6.9-12.4)
	Day 7	180	8.2 (4.9-11.1)	86	8.1 (5.1-12.1)
	Day 28	70	9.4 (6.7-11.2)	31	9.4 (8.2-11.8)
Haematocrit (%)	Day 0	199	26.8 (18.6-35.9)	99	26.2 (18.5-37.1)
	Day 7	179	25.4 (14.7-34.8)	86	25.3 (17.3-37.1)
	Day 28	61	29.1 (23.1-34.8)	29	29.9 (22.8-33.8)
RBC (10^{12} cells/L)	Day 0	199	4.1 (2.4-5.7)	99	4.1 (2.8-5.4)
	Day 7	179	3.8 (2.1-4.8)	86	3.8 (2.1-4.8)
	Day 28	61	4.2 (3.4-5.5)	29	4.3 (3.3-5.5)
WBC (10^9 cells/L)	Day 0	199	10.6 (4.4-37.1)	99	10.3 (5.1-25.9)
	Day 7	178	11.2 (4.2-37.7)	85	11.3 (4.6-25.1)
	Day 28	61	11.2 (5.4-35.5)	29	12.1 (6.4-23.3)
Platelet (10^9 cells/L)	Day 0	199	172.0 (10.0-578.0)	99	150.0 (19.6-576.0)
	Day 7	179	381.0 (30.0-884.0)	86	340.5 (43.0-823.0)
	Day 28	60	358.0 (19.0-529.0)	29	327.0 (41.0-671.0)
Glucose (mmol/L)	Day 0	192	5.9 (4.2-9.5)	97	6.0 ((3.5-8.9)
	Day 7	176	5.3 (3.7-9.8)	85	5.3 (3.0-8.7)
	Day 28	19	5.2 (4.2-7.9)	9	5.7 (5.0-6.8)
ALT (mmol/L)	Day 0	194	19.6 (3.0-140.0)	95	22.0 (1.1-151.1)
	Day 7	172	16.9 (3.0-84.0)	85	18.0 (3.0-90.0)
	Day 28	20	18.5 (7.0-122.0)	8	20.0 (5.0-45.0)
AST (mmol/L)	Day 0	193	46.0 (13.0-229.0)	96	44.0 (9.0-139.0)
	Day 7	174	40.0 (17.0-238.0)	85	39.0 (5.4-189.0)
	Day 28	20	44.0 (27.0-123.0)	9	45.0 (21.0-136.0)
Total bilirubin (µmol/L)	Day 0	192	14.9 (2.0-201.0)	96	15.0 (1.78-128.3)
	Day 7	171	11.0 (2.0-201.4)	85	12.0 (3.0-78.7)
	Day 28	18	7.5 (2.0-17.2)	9	5.0 (2.0-33.0)
Creatinine (µmol/L)	Day 0	193	22.1 (4.4-44.2)	95	22.2 (5.3-81.0)
	Day 7	175	20.5 (7.9-44.2)	83	21.1 (6.0-47.2)
	Day 28	19	20.6 (14.1-44.1)	9	19.6 (12.7-44.2)

RBC, red blood cells; WBC, white blood cells; ALT, alanine aminotransferase; AST, aspartate aminotransferase

Supplementary Table 2. Rules applied for the PCR-Corrected ACPR computation

**Episode occurring during the study**	**Patient classification**	
	**ITT population**	**PP population**
Withdrawal before time of analysis (i.e. day 28 or day 42): any reason except lost to follow-up	Failure	Failure
Withdrawal: reason is lost to follow-up (i.e. patients exit the study for reasons that are not related with treatment)	Failure	Excluded
PCR: non interpretable or missing or not done	Failure	Excluded
PCR: new infection	Success	Success
PCR: recrudescence	Failure	Failure
Early treatment failures (even if treatment course was not completed)	Failure	Failure
